# Cardiovascular Effects Mediated by HMMR and CD44

**DOI:** 10.1155/2021/4977209

**Published:** 2021-07-10

**Authors:** Kinga Jaskuła, Mariusz Sacharczuk, Zbigniew Gaciong, Dominik S. Skiba

**Affiliations:** ^1^Department of Experimental Genomics, Institute of Genetics and Animal Biotechnology, Polish Academy of Sciences, Postępu 36A, Jastrzebiec, Poland; ^2^Faculty of Pharmacy with the Laboratory Medicine Division, Department of Pharmacodynamics, Medical University of Warsaw, Centre for Preclinical Research and Technology, Banacha 1B, Warsaw, Poland; ^3^Department of Internal Medicine, Hypertension and Vascular Diseases, Medical University of Warsaw, Banacha 1A, Warsaw, Poland

## Abstract

Cardiovascular disease (CVD) is the leading cause of death worldwide. The most dangerous life-threatening symptoms of CVD are myocardial infarction and stroke. The causes of CVD are not entirely clear, and new therapeutic targets are still being sought. One of the factors involved in CVD development among vascular damage and oxidative stress is chronic inflammation. It is known that hyaluronic acid plays an important role in inflammation and is regulated by numerous stimuli, including proinflammatory cytokines. The main receptors for hyaluronic acid are CD44 and RHAMM. These receptors are membrane proteins that differ in structure, but it seems that they can perform similar or synergistic functions in many diseases. Both RHAMM and CD44 are involved in cell migration and wound healing. However, their close association with CVD is not fully understood. In this review, we describe the role of both receptors in CVD.

## 1. Introduction

Cardiovascular disease (CVD) is a main cause of death globally, causing an estimated 17.9 million deaths annually. CVD is a general term for a group of heart and blood vessel diseases including coronary heart disease, cerebrovascular disease, and peripheral arterial disease. Late manifestations of CVD are heart attack and stroke mainly caused by previous vascular damage. Chronic inflammation is one of the causes of vascular damage or narrowing [1]. CD44 plays an important role in both inflammation and vascular injury [2]. Inflammation is associated with increased vascular permeability, recruitment of inflammatory cells, and release of inflammatory mediators. The cascade of inflammatory reactions can alter blood flow in the altered tissues by inflammatory cells infiltrating vascular tissues and releasing proteases, cytokines, and reactive oxygen species, which trigger vasoconstriction or relaxation [3], neointimal growth [4], and angiogenesis and tissue remodeling [5, 6]. The main ligand for the CD44 receptor is hyaluronic acid (HA) which binds also to RHAMM (hyaluronan-mediated motility receptor) [7]. HA is regulated by numerous stimuli, including proinflammatory cytokines [8]. Thus, the local production of cytokines within inflammatory lesions in the vessels increases the expression of HA on endothelial cells facilitating CD44-HA interactions and hence causing extravasation of inflammatory cells [9]. The role of CD44 in cardiovascular disease is well described; however, the role of another RHAMM receptor in CVDs is little known. This review discusses the role of both receptors in CVDs and their connections.

## 2. CD44 and RHAMM: Structure and Expression

**Figure 1:**
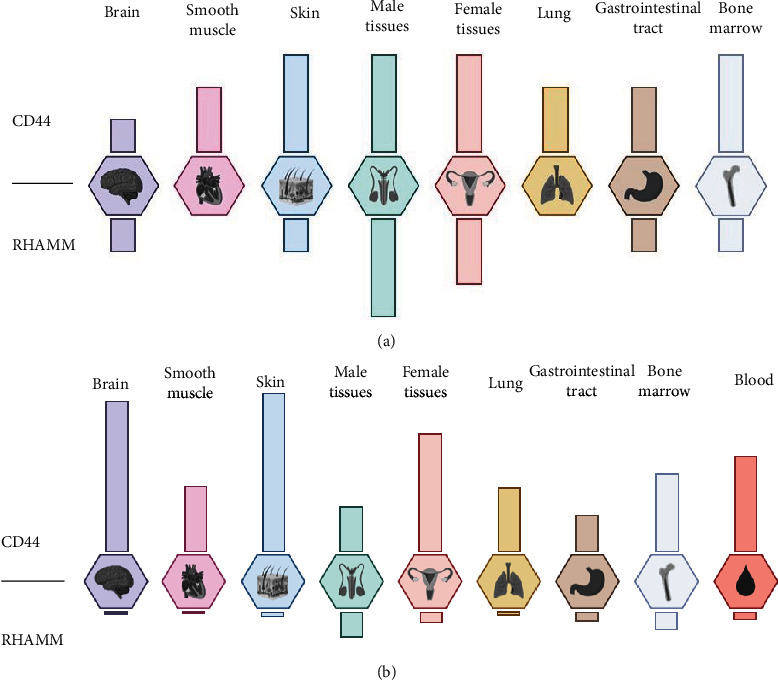
Protein (a) and mRNA (b) expression of CD44. Prepared on the basis of data from the Human Protein Atlas [37]. Protein expression reported with the units: not detected, low, medium, and high. mRNA expression reported as normalized expression (NX) combined from three transcriptomics datasets (HPA, GTEx, and FANTOM5). Created with BioRender.com.

CD44 is a widely expressed cellular adhesion molecule that serves as the major receptor for components of the extracellular matrix (ECM) [10]. Apart from HA, CD44 binds other carbohydrate ligands, such as heparan sulfate [11], as well as noncarbohydrate ligands: collagen and osteopontin [12]. CD44 occurs as a series of isoforms with molecular weights ranging from 85 to over 200 kDa [13]. The most commonly expressed CD44 receptor is the 85-90 kDa glycoprotein which represents the standard CD44 molecule which does not contain products with spliced exon variants [14]. CD44 is structurally and functionally polymorphic. Its gene consists of at least 20 exons, of which 12 can be alternatively spliced. The heterogeneity of the protein is mainly due to the variable splicing of 10 exons encoding the extracellular region located between the invariant HA-binding domain at the NH2 terminus and the membrane proximal extracellular domain [15]. The most common isoform of CD44, called standard form (CD44s), does not contain any exon variants. It consists of a large extracellular domain of 248 amino acids, a 21-amino acid segment encompassing the membrane, and a relatively short cytoplasmic part of 72 amino acids [16]. The *CD44* gene contains 20 exons, of which exons 1-5, 15-17, and 19 encode the CD44 isoform, while exons 6.6a and 7-14 (also designated as vl-v10) are alternatively spliced to generate variant isoforms with insertion into the membrane proximal region of the extracellular domain between amino acids 202 and 203. The amino terminal region is relatively conserved in mammalian species (about 85% homology) and contains a hyaluronan-binding domain, while the membrane proximal region is relatively unconservative (about 35-45% sequence similarity between species) and has several sites for glycosylation and chondroitin sulfate attachment [17, 18]. The transmembrane domain ensures a way to interact with cofactors and adapter proteins and to direct the influx of lymphocytes [19]. The intracellular domain of CD44 has short- and long-tail configurations and performs nuclear localization functions for the regulation of transcription [20].CD44 is expressed in a variety of cell types (Figures 1(a) and 1(b)), including lymphocytes, macrophages, erythrocytes, fibroblasts, neurons, epithelial cells, and endothelial cells [18]. CD44 supports the adhesion of leukocytes to endothelial cells [10], induces the secretion of chemokines from macrophages, and regulates the proliferation and migration of vascular smooth muscle cells [21].

In contrast to CD44, the main hyaluronan receptor, RHAMM, is less well studied and mainly is involved in cell locomotion. It was firstly described by Turley et al. [22, 23] and soon was linked to *ras* transformation and tumor progression [24]. The *HMMR* gene encodes an 85 kDa protein—RHAMM—with an extensive helix structure and a basic globular domain at the amino terminus [25]. It has approximately 35% protein sequence homology to KIF15, a member of the kinesin family [26]. RHAMM does not contain a signal peptide for export via the Golgi apparatus and the endoplasmic reticulum [7]. RHAMM is a member of the hyaladherin protein family, has two hyaluronan-binding domains, and interacts with it through 9–11-amino acid basic motifs [27]. The extracellular part of RHAMM activates signaling cascades that control the expression of cell cycle genes and genes related to cell motility [28, 29]. The intracellular domain of RHAMM is required for the formation of the mitotic spindle and may play a role in the direction of cell motility [30]. The *HMMR* gene is located on the human chromosome 5q33.2 and contains 18 exons [31]. RHAMM, also described as CD168, does not have a transmembrane domain but is anchored by the glycosylphosphatidylinositol (GPI) group in the plasma membrane where it can interact with CD44 and participate in many cell functions, including cell motility, wound healing, and modification of the Ras signaling cascade. Interestingly, RHAMM does not contain a signal peptide and is believed to be transported to the cell surface by unconventional transport mechanisms where it binds to the cell surface by docking to HA synthase [32] and, like CD44, transmits signals affecting cell mobility [27]. It has been shown that RMAMM interacts with hyaluronan in an ionic manner via a 35-amino acid basic C-terminal region, which can be further subdivided into two motifs of 10 and 11 amino acids, respectively [33]. RHAMM is present on the cell surface, in the cytoplasm, and in the nucleus of different types of cells [25] and regulates cell movement and proliferation [24, 25]. RHAMM under physiological conditions is poorly expressed on various cell types (Figures 1(a) and 1(b)) such as lymphocytes, smooth muscle cells, macrophages, and fibroblasts; however, its expression rises in pathological conditions [34, 35].

Although CD44 and RHAMM have different primary amino acid sequences and although CD44 is conservatively expressed in cells and RHAMM is tightly regulated, both receptors possess transforming properties that may be related to their ability to promote motility [34, 36]. Research indicates that during inflammation, wound healing, and tumor formation, cell migration is mediated by CD44 and may require RHAMM surface expression [36]. This means that these receptors may act synergistically in some diseases.

## 3. Atherosclerosis and Vascular Inflammation

Atherosclerosis is an inflammatory disease of the walls of large and medium arteries. Its etiology is not fully understood, but there are several factors influencing its development. Chronic inflammation and increased levels of low-density lipoprotein (LDL) in the blood play a major role in the development of atherosclerosis. Abnormal blood flow in the vessels can cause increased wall tension and promote the production of proteoglycans by arterial smooth muscle cells (SMCs), which can bind and retain lipoprotein molecules, facilitating their oxidative modification, thereby promoting an inflammatory response at lesion sites [38]. Vascular endothelial cells become activated by proinflammatory stimuli and begin to express selective adhesion molecules on the surface, which recruit monocytes and T lymphocytes and which are likely to be involved in the recruitment of blood-borne cells for atherosclerotic lesions [39, 40].

**Figure 2:**
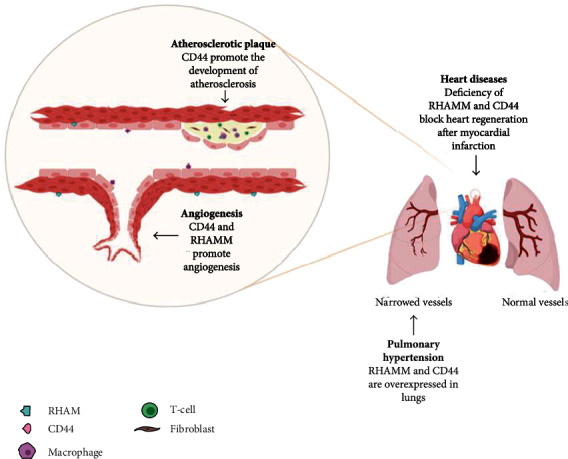
Diagram describing the role of RHAMM and CD44 receptors in the cardiovascular system. Created with BioRender.com.

Atherosclerosis at the developed stage is characterized by the formation of atherosclerotic plaque containing macrophages, dendritic cells (DC), foam cells, lymphocytes, and other inflammatory cells. In advanced age, atherosclerotic plaque calcifications appear [41]. Not only the vascular wall is affected but also the adventitia and adipose tissue attached to the vessel express some degree of inflammation which may precede vascular dysfunction [42, 43]. Dendritic cells and lymphocytes are found in the adventitia and perivascular adipose tissue of normal arteries, but their number is significantly increased in the atherosclerotic arteries [43]. Under internal atherosclerotic lesions, leukocytes organize themselves into clusters resembling tertiary lymphoid tissue [44]. These features of atherosclerotic plaques illustrate that atherosclerosis is a complex disease in which many elements of the vascular, metabolic, and immune systems take part. Atherosclerotic changes result from inflammatory triggers, subsequent release of various cytokines, proliferation of smooth muscle cells, synthesis of the connective tissue matrix, and accumulation of macrophages and lipids [39, 45, 46]. In those processes, CD44 and RHAMM may play an important role (Figure 2).

Numerous studies suggest that the CD44 cell adhesion molecule may promote atherosclerosis by mediating the recruitment of inflammatory cells into platelets and activation of vascular cells [47, 48]. In the atherosclerosis model of apoE^−/−^ mice, vascular expression of CD44 was highest in areas prone to damage [48, 49]. This was confirmed in human studies where CD44 was present in areas of human atherosclerotic plaques, rich in macrophages, and susceptible to rupture, compared to healthy vascular tissues [50, 51]. Elevated expression of CD44 correlated with a 10-fold increase in the secretion of proinflammatory cytokines such as interleukin-1*β* (IL-1*β*) and IL-6 by endothelial cells and macrophages. These cytokines in turn increased CD44 expression [50, 51]. Such a positive feedback loop may exacerbate arteriosclerosis, leading to plaque instability. The elimination of CD44 in mice with the apoE knockout led to a significant reduction in aortic lesions and a reduction in the number of macrophages present in the lesions by 90% [48]. Moreover, gene expression profiling in the aorta of CD44 knockout mice compared to a wild type led to the discovery that CD44 regulates focal adhesion formation, extracellular matrix deposition, and angiogenesis, critical processes for atherosclerosis [49]. To investigate the mechanism by which CD44 controls atherosclerosis, bone marrow chimeras were generated using bone marrow transplant from a wild type (WT) and CD44-null donor to apoE^−/−^ and apoE^−/−^ CD44^−/−^. The expression of CD44 in both the vascular and bone marrow cells contributed to the development of changes in the apoE^−/−^ model. It means that CD44, on both the resident and recruited cells, is essential for its full proatherogenic effect *in vivo* [47]. Moreover, the CD44 deletion also favored an increase in fibrotic lesions in apoE^−/−^ CD44^−/−^ mice compared to apoE^−/−^ mice, which indicates that CD44 also regulates the lesion composition and influences the stability of atherosclerotic plaques [47].

So far, RHAMM has not been directly associated with atherosclerosis, but it is known that it plays a vital role in inflammation, an important factor in the pathogenesis of atherosclerosis [36]. It has been shown that RHAMM interacts with growth factor receptors such as PDGFR [52], TGF-*β* receptor I [53], or bFGFR [54]. Growth factors regulate the function of ERK signaling and are responsible for the regulation of cell proliferation and differentiation [52]. In addition, ERK signaling has been shown to play a role in altering cholesterol homeostasis in human macrophages [55]. By interacting, RHAMM takes part in the mobility necessary for the inflammation by activating ERK1/2/MAPK. Nuclear RHAMM is also associated with the ERK1/2/MAP kinase, which mediates the activation of PAI-1 and MMP-9, which are involved in cell mobility and inflammation [56]. RHAMM also participates in HA-dependent regulation. HA is produced during tissue damage, causing activation of inflammatory cells to induce innate immune response and regulation of the behavior of epithelial cells and fibroblasts [57–59]. Additional confirmation of the importance of RHAMM is the fact that the use of a 15-mer peptide with homology to RHAMM-binding sequences has been shown to block HA signaling and reduce inflammation and fibrogenesis [60].

Macrophages in atherosclerotic plaque formation play a fundamental role [61]. Macrophages colonizing the atherosclerotic plaque have a reduced migration capacity, which leads to the maintenance of inflammation and further progression into the atherosclerotic plaque [62]. Moreover, they participate in the intake and accumulation of lipoproteins. Cholesterol uptake by macrophages leads to their transformation to foam cells in the vascular wall. At this stage of atherosclerosis, fatty streaks are observed in the vasculature. The RHAMM receptor is expressed on macrophages. In a rat model of acute lung damage, the expression of RHAMM and HA is increased in macrophages responding to intratracheal injury [63, 64]. Moreover, *Hmmr*^−/−^ transgenic mice have been shown to exhibit reduced macrophage chemotaxis [65]. Another element essential in the development of atherosclerotic lesions includes vascular smooth muscle cells (VSMCs). VSMCs, under the influence of vascular damage, are able to change the quiescent “contractile” phenotype into a “proinflammatory” phenotype. Activated VSMCs can effectively multiply and migrate, helping to repair vascular walls. However, in the chronic inflammation that occurs in atherosclerosis, VSMCs are misregulated, leading to extracellular matrix formation in the plaque areas [66]. Research has shown that RHAMM can be an important element in these processes. *In vitro* studies have shown that HA mediates VSMC migration through CD44 and RHAMM receptors as well as VSMC proliferation but only through the CD44 receptor [2, 67, 68]. On the basis of the culture of bovine vascular smooth muscle cells, an increase in RHAMM expression was observed after scratch wound assay. It was concluded that vascular damage leads to an increase in RHAMM expression and is localized in the VSMC at the edge of the lesion [68].

## 4. Ischemic Heart Disease

Ischemic heart disease is one of the most common causes of death in developed countries. After myocardial infarction (MI), billions of cardiomyocytes undergo apoptosis, pyroptosis, and necrosis, resulting in a noncontractile collagen scar that reduces heart function [69]. The mammalian heart's ability to replace lost cardiomyocytes is limited, while adult zebrafish (*Danio rerio*) can successfully regenerate the heart following apical ventricular amputation throughout its lifetime [70, 71]. A key factor in the regeneration process in zebrafish is the ability of preexisting cardiomyocytes to proliferate after organ damage [72–74].

After analyzing the proteomic changes following ventricular apex resection in adult zebrafish, increased expression of the RHAMM was identified. It was also investigated that after zebrafish ventricular resection, the area of scar tissue was significantly larger in the RHAMM knockdown fish, suggesting that the RHAMM knockdown blocked heart regeneration. The importance of hyaluronic acid in the regeneration of the heart was also determined. It was found that after inhibition of HA synthesis, after ventricular resection in zebrafish, significant scar tissue was still present compared to minimal or no scar tissue in controls [75].

Studies on the role of CD44 in regeneration after MI have shown a correlation between inflammatory mediators and CD44 in regulating the inflammatory response, repairing the heart, and differentiating the heart fibroblasts after MI [76]. Studies in CD44-deficient mice that underwent myocardial infarction showed prolonged inflammation, reduced collagen deposition in scars, decreased myofibroblast infiltration, and decreased TGF-*β* signaling [77]. Following acute MI, IL-6 has been shown to enhance CD44 and HA synthase (HAS-1 and HAS-2) expression in cardiac fibroblasts, resulted in a matrix rich in HA. As a result, proinflammatory cytokines and the expression of smooth muscle *α*-actin were induced through CD44-HA interactions. This interaction, together with circulating IL-6, changed the nature of the ECM, modulated the differentiation of cardiac fibroblasts, and promoted the immune responses [78].

## 5. Vascular Remodeling

Restrictive remodeling assumes that SMCs reorganize the ECM, while the factors causing constrained remodeling compared to external remodeling remain poorly defined [79]. The contraction of the vessel wall is similar to that of a cutaneous wound. SMCs repopulate the sites of vascular damage and cause the production and remodeling of the ECM, which changes the geometry of the vessel wall. It is known that matrix remodeling depends in part on direct adhesion interactions of ECM cells. In *in vitro* studies on SMCs, HA consistently increased the adhesion of SMCs to collagen-precoated plates and blocking or removing RHAMM weakened SMC adhesion to collagen with or without exogenous HA [80]. That suggests that the endogenous production of HA is sufficient to activate RHAMM. RHAMM activation by HA significantly influences the adhesive interactions between SMC and ECM and contributes to remodeling of the wall and narrowing of the lumen after carotid artery ligation [80]. RHAMM-blocking strategies at sites of vascular injury may be potentially useful in the prevention of clinical restenosis.

The role that CD44 plays in remodeling, for example, after angioplasty, is unclear. Available data published on that topic does not clearly indicate whether CD44 may be a remodeling factor or may play a protective role by inhibiting neointimal formation [81, 82]. However, studies have shown that the deficiency of the *CD44* gene significantly enhanced neointimal hyperplasia, which suggests that the *CD44* gene is involved in the process of pathological remodeling and may play a protective role. The remodeling response to injury involves circulating cells that arise from the bone marrow as well as cells from the local artery wall. Compared with damaged femoral arteries in CD44^+/+^ mice, CD44^−/−^ mice showed significantly greater femoral vascular remodeling. In remodeled femoral arteries from CD44^−/−^ mice, no significant changes were observed in CD44 expression when compared to intact arteries. After the use of low-mass weight (LMW) heparin, the damaged arteries showed a significant reduction in neointimal thickness and a significant increase in CD44 expression. This suggests that CD44 may be the route by which LMW heparin diminishes the remodeling process [83].

## 6. Angiogenesis

Angiogenesis is the formation of new blood vessels from existing vessels (Figure 2). It can occur in a pathological form in the context of circulatory diseases or cancer and in a physiological form in the case of tissue ischemia, wound healing, etc. [84]. To investigate the involvement of CD44 in blood vessel formation, *in vivo* angiogenesis was studied in CD44-deficient mice [85]. Initial studies were performed in a model in which vessels develop around and within subcutaneously implanted Matrigel plugs containing B16 murine melanoma tumor cells as a source of angiogenic growth factors. Vascularization of the plugs was observed in wild-type animals, but not in CD44 knockout mice, confirming the involvement of CD44 in the pathological formation of blood vessels [86]. In addition, in a mouse oral carcinogenesis model, after implantation of Matrigel plugs with suspended cancer cells into the dorsal chamber of the skin fold, CD44^+/+^ mice showed a higher microvascular density than CD44^−/−^ [87]. It has also been shown that inhibition of CD44 reduces the adhesion of endothelial cells (EC) to HA immobilized on the plastic surface [54]. This means that an increase in CD44 expression may allow the adhesion of the EC to the components of the ECM, which is one of the processes enabling the formation of new blood vessels [1]. Whether CD44 plays a role in physiological angiogenesis has also been investigated. Wound closure in animals with the CD44 knockout was shown to be delayed within 1 to 3 days after wounding, relative to wild-type animals. Vascular density at the edge of the wounds on day 3 was reduced by around 20% in CD44^−/−^ animals compared to wild-type mice. This means that the lack of CD44 results in an early delay in skin wound closure, which is associated with reduced neovascularization of the injured tissue. The above results thus confirmed that CD44 may be involved in physiological angiogenesis [86]. The differentiation and organization of EC in blood vessels is a critical step in angiogenesis [88]. To investigate the role of RHAMM receptors in blood vessel formation, the effect of anti-RHAMM antibodies on EC function and *in vivo* angiogenesis was determined. Using the endothelial tube formation model on Matrigel, anti-RHAMM antibodies were found to inhibit tube formation by human endothelial cells. It was also investigated that the use of an anti-RHAMM antibody in a mouse model of angiogenesis significantly reduced the vascularization of the plugs [54].

## 7. Idiopathic Pulmonary Arterial Hypertension

Idiopathic pulmonary arterial hypertension (IPAH) is a disorder characterized by persistent elevated pulmonary arterial pressure with unknown causes [89]. The primary mechanism of IPAH is pulmonary vascular remodeling involving pre- and intra-acinar arteries, such as stenotic lesions and complex lesions characterized by plexiform lesions (Figure 2) [90]. The plexiform injury has been studied in relation to idiopathic pulmonary arterial hypertension as a marker of the severity or rapid progression of pulmonary hypertension [91], but it also contributed to the pathogenesis of the disease. Studies have shown that CD44 was frequently expressed in plexiform lung lesions in IPAH patients and was mainly located in endothelial cells that make up the microvasculature of the lesions and surrounding T lymphocytes. However, CD44 was not found in any of the vascular cells of normal pulmonary arteries. This suggests that CD44 is involved in the pathogenesis of idiopathic pulmonary arterial hypertension [92]. The role of RHAMM in pulmonary hypertension is not fully understood. However, studies have shown that loss of PPAR*γ* (peroxisome proliferator-activated receptor *γ*) is associated with pulmonary hypertension [93]. It has been shown that PPAR*γ* expression is significantly reduced in plexiform lesions in humans with PH. Reduced PPAR*γ* expression was also demonstrated in vascular lesions in a rat model of severe pulmonary hypertension [94]. It has also been investigated that by pharmacological inhibition of PPAR*γ*, RHAMM was upregulated in pulmonary arterial endothelial cells in a sheep model of pulmonary hypertension. The increased expression of RHAMM was also confirmed in human pulmonary microvascular endothelial cells (HMVEC) with PPAR*γ* depletion. These results suggest that HMMR plays a large role in hypertension but it would be worthwhile to conduct more research in this area [95].

## 8. Conclusions

CD44 and RHAMM mediate different cardiovascular effects in normal and pathological conditions. Herein, we discussed the role of these receptors not only in cardiovascular diseases such as atherosclerosis, pulmonary hypertension, and ischemic heart disease but also in cardiovascular processes such as vascular inflammation, vascular remodeling, and angiogenesis. Expression of RHAMM in contrast to CD44 is relatively low in vascular cells and blood. However, RHAMM expression rises in pathological conditions. Expression of CD44 was found to be upregulated in areas prone to atherosclerosis. The important role of RHAMM in atherosclerosis development is its role in the motility of the immune cells. The gained ability of migration mediated by RHAMM allows cells to the inflammatory response. This process is important also in other conditions where vascular remodeling is present. In conditions after myocardial infarction, CD44 and RHAMM help in wound healing by reorganization of the extracellular matrix and collagen deposition. Inhibition or lack of RHAMM or CD44 blocks angiogenesis in both the physiological and pathological conditions. Summarizing the role of both described receptors is similar apart from its different role in vascular remodeling. However, the RHAMM receptor is significantly less studied in cardiovascular diseases.
